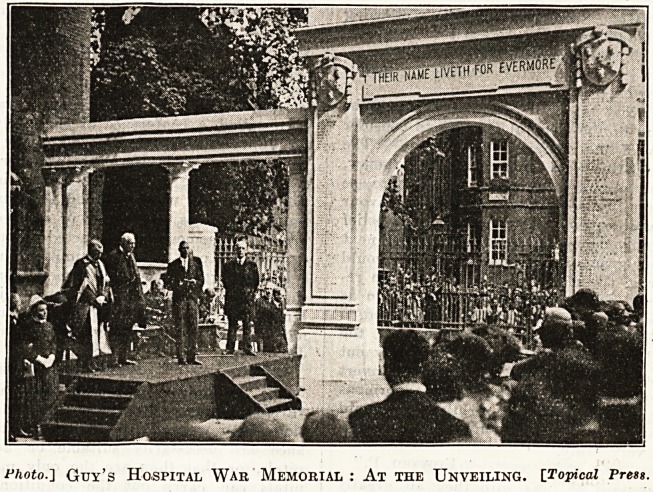# Unveiling by the Duke of York

**Published:** 1921-07-23

**Authors:** 


### GUY'S HOSPITAL WAR MEMORIAL.

### Unveiling by the Duke of York.

THE amount of time which the Prince of Wales spends in the service of the hospitals is, we think, hardly realised. To take but one week since his return from Lancashire, a week during which H.R.H.'s regretted indisposition has necessitated the cancelling of all his engagements. On Wednesday morning, July 13, he was to have attended an important meeting of the Council of King Edward's Hospital Fund, of which he is President and to the affairs of which he devotes the closest attention. On the same afternoon he, with Princess Alice, Countess of Athlone, had arranged to receive the guests at the League of Mercy garden-party at St. James's Palace; on Friday, July 15, he had promised to unveil the war memorial to the students of Guy's Hospital, of which also he is President, and to open the new massage building; while on Tuesday, July 19, he was due in Sheffield, where he would have paid a visit to the Royal Infirmary, the first Royal visit by the way that this institution would have received, though it was granted the designation of Royal so long ago as 1897. This record of H.R.H.'s intended activities for one week in the cause of hospitals is one of which the nation and the hospitals may well be proud.

The Prince of Wales's ready sympathy in the cause of the suffering is shared by his brother, the Duke of York, who at very short notice consented to fill the Prince's place at Guy's on Friday, July 15. His Royal Highness, who was attended by Wing-Commander Greig, was received by Viscount Goschen, the treasurer of the hospital, and, after a brief ceremony in the Court Room, when he was elected a Governor of the hospital, was conducted through the quadrangle lined with members of the nursing and domestic staffs, and some convalescing patients to a daïs in the "Park." With him were Viscount Goschen, Dr. Fawcett, Mr. H. Cosmo Bonsor, and Sir Alfred Fripp. Mr. BONSOR, as chairman of the War Memorial Committee, read an address, which, like the speeches following, must have been inaudible to all owing to the clamorous enthusiasm of the children of the district, who thronged Great Maze Pond immediately adjacent to the Memorial. But this was the only blemish in the afternoon's arrangements, and it is one which lovers of children will readily forgive. Mr. BONSOR, after expressing regret at the cause of the Prince of Wales's absence, hopes for his speedy recovery, welcome to the Duke of York as a Governor of Guy's and gratitude for his presence that day at such short notice, gave some particulars of the expenditure of the £5,300 collected for a War Memorial. £3,000 has been vested in trustees to provide assistance, if necessary, to the dependants of those Guy's men who fell in the war and to form a permanent War Memorial Scholarship for the relations of those who lost their lives or were disabled, who should have preferential claims in the examination to obtain it. The sums allocated to these two objects are £1,000 and £2,000 respectively. The first student has already been elected to the scholarship. The balance has been applied as to £2,000 in erecting the Memorial and as to £300 for the memorial adornment of the college dining-hall.

The permanent Memorial is in the form of a beautiful stone archway flanked on either side by a pillared screen, the design being that of the hospital architect, Mr. Walford. The names of those who died during the war are carved upon the two pillars of the main arch, and above the archway are engraved the words, "Their name liveth for evermore." On the reverse side are the words, "Erected by the friends of the Guy's Men who gave their lives in the Great War, 1914-1919." The motto of the hospital, "Dare quam accipere," also appears on the Memorial, which stands just within the Great Maze Pond entrance to the hospital, facing the Students' Residential College and Club. Under the arch of this Memorial all Guy's students, both now and hereafter, will pass. Mr. Bonsor's concluding words in his address on Friday were a simple and fitting tribute to the memory of those who gave their lives:

*Photo.*] GUY'S HOSPITAL WAR MEMORIAL: AT THE UNVEILING. [*Topical Press.*

"We have erected this archway, with their names attached, in order that their memory shall always be with us, reminding us of their devotion and sacrifice and inspiring us to follow their example of putting their country's good before personal ambitions." Dr. FAWCETT, senior physician of the hospital, following Mr. Cosmo Bonsor, said, in addressing the Duke of York: "All over this Empire of ours, Guy's men, their parents and sons, will thank you for the kindly thought and gracious act by which you to-day record in public your sympathy with us at Guy's in the loss of the men whose names are engraved on these columns, and who, giving up their lives 'for King and Country,' have exemplified in full the meaning of our motto of 'Dare quam accipere.' May we, who pass under this arch in future, never forget what we owe to them, and play our small part in life the better for the way they played theirs."

His Royal Highness then unveiled the Memorial, and, returning to the dais, said: "My brother the Prince of Wales wishes me to say how very deeply he regrets that his temporary indisposition has prevented him from being here this afternoon to perform this important ceremony. In his unavoidable absence it has been my very great privilege to unveil this Memorial to commemorate the gallant men of Guy's Hospital who gave up their lives during the Great War, and I would like to take the opportunity of expressing my sincere sympathy with the relatives and friends of those whose names figure upon your very long roll of honour."

Proceeding to the new massage building, which was described and illustrated in last week's issue, H.R.H. inspected and formally opened it; and then, standing on the steps, made a short impromptu speech, thanking the guests for the welcome given to him, and Lord Goschen and the Governors of the hospital for having elected him a Governor that afternoon. He concluded, "I am very proud indeed of being associated with Guy's." Rousing cheers were given for His Royal Highness, who then departed.

We believe that the form of Guy's Hospital War Memorial will commend itself to many other institutions which desire to commemorate their fallen. In its memorial arch, its assistance of dependants, and its endowment of scholarships, it combines beauty and imagination, permanence, and distinctiveness of object, which make it a worthy memorial to the 1,788 Guy's men who served during the war, of whom 128 were killed in action or died of disease or wounds. No fewer than 265 Guy's men received honours.

## Figures and Tables

**Figure f1:**